# Challenges in the Treatment of HIV-Related Lymphomas Complicated by COVID-19: Case Study and Review of the Literature

**DOI:** 10.3390/ph18101461

**Published:** 2025-09-28

**Authors:** Kinga Siewiorek, Marcin Jasiński, Błażej Izdebski, Maciej Przybylski, Małgorzata Kobylecka, Joanna Mączewska, Krzysztof Jamroziak, Joanna Drozd-Sokołowska

**Affiliations:** 1 Department of Hematology, Transplantation and Internal Medicine, Medical University of Warsaw, Banacha 1a Str., 02-097 Warsaw, Poland; 2 Doctoral School, Medical University of Warsaw, 02-091 Warsaw, Poland; 3 Chair and Department of Medical Microbiology, Medical University of Warsaw, Chałubińskiego 5 Str., 02-004 Warsaw, Poland; maciej.przybylski@uckwum.pl; 4 Department of Nuclear Medicine, Medical University of Warsaw, Banacha 1a Str., 02-097 Warsaw, Poland

**Keywords:** HIV-related lymphomas, diffuse large B-cell lymphoma, SARS-CoV-2, AIDS, immunocompromised patient, molnupiravir, shedding duration

## Abstract

Lymphomas remain a significant cause of morbidity and mortality among patients living with HIV. Although the introduction of antiretroviral therapy has led to a reduction in the incidence of AIDS-related lymphomas (ARL) and an overall improvement in prognosis, these malignancies continue to pose a considerable clinical challenge. Beyond the inherent complexity of lymphoma treatment itself, the management of comorbidities, particularly infections, represents a therapeutic obstacle. Here, we review the published evidence on ARL complicated by COVID-19. Despite the fact that nearly 800 million confirmed cases of SARS-CoV-2 infection have been reported so far, only five cases of ARL and COVID-19 have been published, among whom most patients experienced a mild course of SARS-CoV-2 infection, with only one case progressing to severe COVID-19 that required oxygen therapy and prolonged hospitalization. Additionally, we present another case of a 49-year-old male patient with newly diagnosed ARL, Epstein–Barr virus (EBV)-positive, diffuse large B-cell lymphoma, not otherwise specified, complicated by prolonged SARS-CoV-2 infection. Although initially asymptomatic, the patient subsequently experienced transient respiratory failure. Despite administration of molnupiravir, both SARS-CoV-2 antigen and RT-qPCR tests remained positive for a minimum of 113 days. The prolonged SARS-CoV-2 infection, in conjunction with other opportunistic infections, impeded the delivery of adequate chemotherapy dose intensity and contributed to disease progression and ultimately the patient’s death. This case and review of the literature underscores the diversity of the clinical course of SARS-CoV-2 infection in patients with ARL and highlights the associated challenges in delivering optimal anti-lymphoma therapy in those patients.

## 1. Introduction

Since the first reports of pneumonia in Wuhan, China, at the end of 2019, SARS-CoV-2, the virus that causes coronavirus disease 2019 (COVID-19), has spread to all continents. It has contributed to more than 778 million confirmed cases, including over 7 million deaths, as reported by the World Health Organization (WHO) (data as of 13 July 2025) [1]. A group of patients at increased risk of a severe course of COVID-19, with a more frequent requirement for intensive care unit admission and higher mortality, comprises those with hematological malignancies, as demonstrated in numerous studies [2,3,4,5,6,7,8]. Moreover, in these patients, a delay between the onset of symptoms and clinical deterioration is common; the viral load is higher, and the median duration of viral test positivity is prolonged, compared to the general population [2]. Prolonged viral shedding, which can persist for several months [9,10,11], has also been reported in other immunocompromised populations, including solid organ transplant recipients [12], patients receiving anti-CD20 therapy [4,12], and individuals living with human immunodeficiency virus (HIV) [11,12,13]. Although substantial knowledge has been gained regarding the clinical course, transmission mechanisms, and pathogenesis of SARS-CoV-2, data concerning patients with hematological malignancies, particularly HIV-related lymphomas (ARL), remain significantly limited. To the best of our knowledge, only a few individual cases of SARS-CoV-2 infection in people living with HIV (PLWH) diagnosed with lymphoma have been reported to date [11,14,15,16] (Table 1). Here, we summarize those cases and present an additional case of newly diagnosed acquired immunodeficiency syndrome (AIDS)-related, Epstein–Barr virus (EBV)-positive, diffuse large B-cell lymphoma (DLBCL), not otherwise specified (NOS), with persistently positive antigen and reverse-transcription real-time polymerase chain reaction (RT-qPCR) SARS-CoV-2 test results for a minimum duration of 113 days.

## 2. Case Report

A 49-year-old Korean male residing in Poland, with no significant past medical history, was admitted to the hospital in December 2021 due to suspected severe ulcerative colitis. The patient reported increasing pain in the left iliac fossa, along with diarrhea persisting for the preceding three months, intermittently mixed with blood, as well as fatigue and weight loss. Prior to admission, he had undergone an outpatient abdominal computed tomography (CT) scan, which revealed thickening of the large intestinal wall. Colonoscopy demonstrated inflammatory lesions involving the entire colon and multiple ulcers.

Upon admission, physical examination revealed cachexia, abdominal rigidity with tenderness in the left lower quadrant, and tachycardia. Laboratory investigations were notable for microcytic anemia, with a hemoglobin concentration of 10.2 g/dL (reference: 14–18 g/dL), mean corpuscular volume (MCV) of 77.5 fL (80–97 fL), lymphocytopenia of 0.9 × 10^9^/L (1–4 × 10^9^/L), hypoalbuminemia of 22 g/L (35–53 g/L), and elevated C-reactive protein (CRP) at 34.3 mg/L (0–10 mg/L). *Clostridioides difficile* infection was excluded. An abdominal X-ray revealed no abnormalities. Sigmoidoscopy showed areas of pale mucosa with diminished vascular pattern in the rectum. The sigmoid mucosa was focally covered with blood and clots, with a single ulcer and a colonic polyp also identified. Standard treatment for ulcerative colitis, including corticosteroids and mesalazine, was initiated. On the following day, the patient developed severe abdominal pain, rigidity, tenderness with guarding, and absence of bowel sounds. Repeat abdominal X-ray revealed gastrointestinal perforation. An emergency laparotomy was performed, during which a fistula between the jejunum and sigmoid colon, along with perforation at that site, was identified. Segmental resection of the small intestine, resection of the sigmoid colon, and an end-to-end descending-sigmoid anastomosis were performed.

The patient was initially treated with empirical broad-spectrum antibiotics, which were later adjusted based on intraoperative culture results indicating *Enterobacter cloacae*. The postoperative course was complicated by intra-abdominal bleeding. A re-laparotomy revealed active bleeding from the mesentery at the site of sigmoid colon resection and leakage at the descending-sigmoid anastomosis. Hemostasis was achieved intraoperatively, the anastomosis was taken down, and an end colostomy was constructed. One week later, the patient experienced hematemesis accompanied by a drop in hemoglobin concentration. Gastroscopy revealed grade D esophagitis according to the Los Angeles Classification [18]; additionally, a vessel covered with a fresh clot was identified in the fundus, and a hemostatic clip was applied.

**Figure 1:**
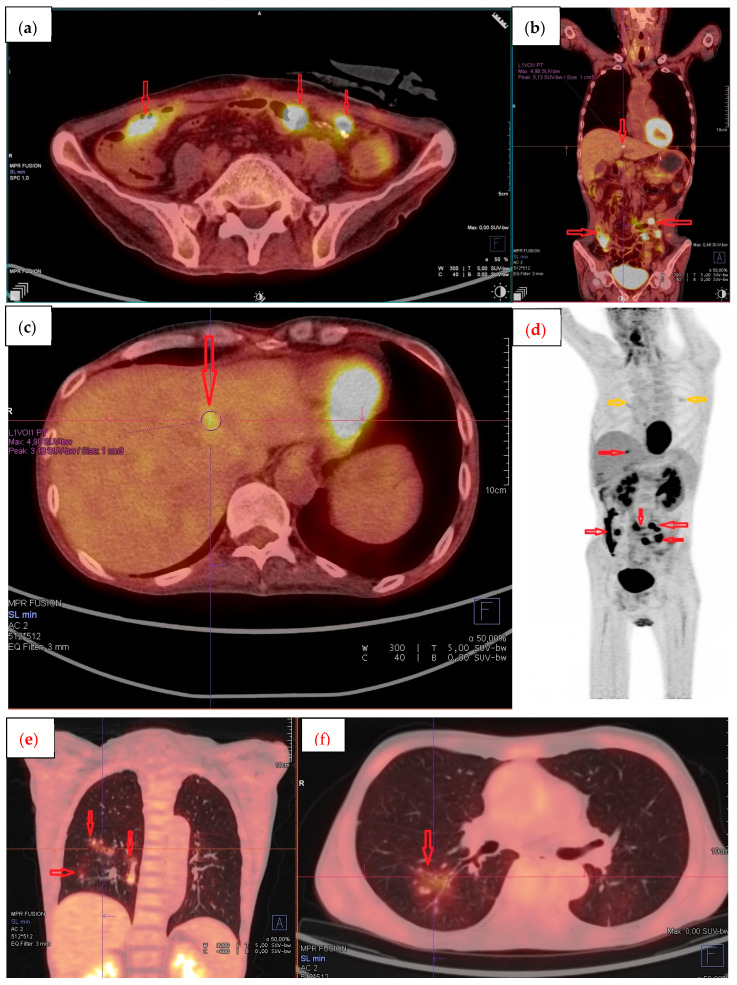
Baseline [^18^F] FDG PET/CT scans before initiation of immunochemotherapy. (**a**) Axial image showing high tracer uptake in pathological peritoneal and intestinal infiltrations (red arrows). (**b**) Coronal image with pathological peritoneal and intestinal infiltrations (red arrows). (**c**) Axial image showing focal tracer uptake in the liver due to lymphoma (red arrow). (**d**) Coronal image with high tracer uptake in pathological peritoneal and intestinal infiltrations (red arrows); inflammatory changes in the lungs (yellow arrows). (**e**,**f**) Axial and coronal images showing diffuse tracer uptake in the lungs due to inflammatory changes (red arrows).

The final diagnosis was established based on the histopathological examination of resected samples from the small intestine and sigmoid colon. The findings did not support the initial diagnosis of ulcerative colitis but instead revealed infiltration by large, polymorphic lymphoid cells with the following immunophenotype: CD20+, PAX5+, CD3−, CD5−, BCL2+, c-MYC−, CD10−, BCL6+, MUM1+, CD30+, Cyclin D1−, and ALK1−. The proliferative index (Ki-67) was 70%. The neoplastic cells were diffusely positive for Epstein–Barr virus encoding region (EBER). In addition, histopathological features consistent with cytomegalovirus (CMV) colitis were observed. The final diagnosis was EBV-positive diffuse large B-cell lymphoma (DLBCL), not otherwise specified (NOS), according to the WHO 2016 classification [19]. Positron emission tomography/computed tomography (PET/CT) revealed increased uptake of [^18^F] fluorodeoxyglucose (FDG) in the mesentery, peritoneum, small intestine, rectal wall, and liver, with maximum standardized uptake values (SUVmax) reaching up to 30 (Figure 1). Involvement of the bone marrow and central nervous system (CNS) was excluded. The disease was classified as stage IV according to the Lugano classification [20] with prognostic indices: international (IPI) [21] of 3 points (Eastern Cooperative Oncology Group (ECOG) Performance Status 3, clinical stage IV, extranodal involvement > 1 site), and age-adjusted (aaIPI) of 2 points.

Before the initiation of anti-lymphoma therapy, standard virological testing was performed, revealing a broad spectrum of viral infections, including HIV, cytomegalovirus (CMV), Epstein–Barr virus (EBV), and past hepatitis B virus (HBV) infection. The patient tested positive for HIV in both screening and confirmatory assays, with an HIV RNA load of 562,800 copies/mL and a CD4+ T lymphocytes of 16 cells/μL. CMV DNA was detected in plasma at 7.6 × 10^5^ copies/mL. EBV DNA levels in serum were low. Serological testing showed positive anti-HBc antibodies without detectable HBV DNA. Chest X-ray demonstrated interstitial infiltrates in both lungs, suggestive of *Pneumocystis jirovecii* pneumonia (PjP) or CMV pneumonitis. Ophthalmological examination confirmed CMV retinitis. Combined antiretroviral therapy (bictegravir/emtricitabine/tenofovir alafenamide) was initiated, along with treatment for opportunistic infections: CMV gastrointestinal disease, retinitis, and pneumonitis (valganciclovir, later replaced by ganciclovir), and PjP (trimethoprim-sulfamethoxazole with adjunctive prednisone). Antifungal prophylaxis with fluconazole and empirical antibiotic therapy were also administered. Additionally, due to hypogammaglobulinemia, polyclonal intravenous immunoglobulins (IVIG) were given. The patient was subsequently qualified for immunochemotherapy with the R-CHOP regimen (rituximab, cyclophosphamide, doxorubicin, vincristine, prednisone). The first cycle was administered without rituximab due to positive anti-HBc serology and ongoing HBV diagnostic evaluation. Intrathecal triple chemotherapy (methotrexate, cytarabine, dexamethasone) was also administered as central nervous system (CNS) prophylaxis. Five days after the first CHOP cycle, routine nasopharyngeal swab testing returned positive for SARS-CoV-2 on RT-qPCR (Table 2). At that time, SARS-CoV-2 screening was performed in our hospital on a weekly basis in immunocompromised patients receiving chemotherapy, regardless of symptoms. Notably, the patient had been fully vaccinated against SARS-CoV-2, and all prior screening tests, including one on admission and subsequent weekly tests, had been negative. Although asymptomatic at the time, the patient received a five-day course of molnupiravir due to the high risk of developing severe COVID-19.

Before the second chemotherapy cycle, a follow-up CT scan showed no new pulmonary infiltrates. Partial radiologic response to DLBCL treatment was observed. However, persistent SARS-CoV-2 antigen positivity led to a temporary delay in chemotherapy.

Given the high risk of lymphoma progression and the patient’s ongoing asymptomatic SARS-CoV-2 infection, despite continued RT-qPCR positivity on day 24, the second CHOP cycle was administered. Rituximab was again omitted due to active SARS-CoV-2 infection. This cycle was complicated by febrile neutropenia, despite primary prophylaxis with granulocyte colony-stimulating factor (G-CSF). Clinical deterioration followed, including elevated inflammatory markers and eventual respiratory decompensation. From day 39, a progressive decline in blood oxygen saturation was observed. At that time the patient was no longer receiving specific antiviral treatment for SARS-CoV-2, having completed a 5-day course of molnupiravir earlier. Supportive care and antimicrobial therapy for bacterial and fungal coinfections were ongoing. Initially, the patient required low-flow nasal oxygen therapy (6 L/min), but by day 41, he developed dyspnea with oxygen saturation dropping to 69%. High-flow nasal cannula (HFNC) oxygen therapy was initiated. Chest high-resolution computed tomography (HRCT) revealed obstruction of the left main bronchus, resulting in total atelectasis of the left lung. Bilateral pleural effusions were also noted. Bronchoscopy was performed, and residual bronchial secretions were aspirated, restoring full function of the left lung.

Microbiological cultures from sputum and bronchoalveolar lavage fluid (BALF) grew *Acinetobacter baumannii* and *Candida glabrata*. Targeted therapy with colistin and caspofungin was initiated based on susceptibility profiles. During this period, both SARS-CoV-2 antigen and RT-qPCR tests remained positive, with decreasing cycle threshold (Ct) values (Table 2).

At that time, patient developed recurrent gastrointestinal bleeding and was diagnosed with pseudomembranous colitis in the context of active CMV infection, evidenced by rising CMV DNA levels. Treatment included vancomycin, metronidazole, and foscarnet, the latter due to suspected ganciclovir resistance.

With gradual improvement in respiratory status, HFNC oxygen therapy was discontinued on day 66, and by day 74, no further oxygen supplementation was required. A CT scan following two CHOP cycles showed bilateral ground-glass opacities in the lungs and radiological evidence of DLBCL recurrence. This included multiple hypodense hepatic lesions and a 65 × 60 mm mass in the lower left abdomen infiltrating the abdominal wall and possibly the small intestine. Histopathological confirmation of lymphoma recurrence was obtained.

Given the patient’s poor general condition, disease relapse, persistent neutropenia after the second CHOP cycle, multiple coexisting infections, and the inability to continue full-intensity lymphoma treatment, the decision was made to refer the patient to hospice care. While awaiting transfer, the patient developed sepsis due to *Acinetobacter baumannii* and *Enterococcus faecalis* and died on day 132 of prolonged SARS-CoV-2 infection.

## 3. Review of the Literature and Discussion

Coexistence of HIV infection, lymphoma and COVID-19 is a rare situation. Although each of these three conditions is commonly treated individually, their simultaneous occurrence has been rarely studied, with only few case reports published to date [11]. The coexistence of these conditions presents a significant challenge for treating physicians, particularly regarding the appropriate selection and timing of chemotherapy or chemoimmunotherapy. It also creates logistical difficulties when a patient with an active SARS-CoV-2 infection continues to test positive in the post-pandemic era, during which dedicated COVID-19 treatment facilities are no longer available.

As mentioned already, to date, only five cases of SARS-CoV-2 infection in PLWH diagnosed with lymphoma have been reported in the available literature (Table 1). When comparing our case with the previously published reports summarized in Table 1, several important differences emerge. Most of the reported patients with HIV-related lymphoma and concomitant SARS-CoV-2 infection experienced a mild course of COVID-19 [11,15,16]. In those cases, anti-lymphoma treatment could be administered either concurrently (R-CHOP in the case described by Maan et al. [11]) or shortly before COVID-19 diagnosis, without apparent worsening of the viral illness. One patient described by Kuczborska et al. [15] received radiotherapy during active infection and also had only a mild course of COVID-19. In contrast, our patient developed prolonged SARS-CoV-2 infection with persistently positive antigen and RT-qPCR results for at least 113 days, accompanied by multiple opportunistic and nosocomial infections, ultimately leading to an inability to deliver optimal chemotherapy dose intensity.

A more severe course of COVID-19 was reported in two cases: Szwebel et al. [14] described a patient with B-cell lymphoma and prior exposure to multiple lines of therapy including anti-CD20 monoclonal antibodies, who developed respiratory failure and required advanced COVID-19-directed therapies; Arbune et al. [17] presented a patient with Hodgkin lymphoma and hypoalbuminemia who also developed severe COVID-19. However, in both of those cases, the patients had stable HIV infection with undetectable viral load. Our case differs significantly as the patient had newly diagnosed AIDS with profound immunosuppression (CD4 count 16/µL), which likely contributed to both prolonged viral shedding and poor outcome.

We present another case of a patient with AIDS-related DLBCL and concomitant COVID-19. In the presented case, we encountered the issue of prolonged SARS-CoV-2 infection, which posed a substantial obstacle to the effective treatment of the hematological malignancy. In the general population of patients with hematological malignancies and concurrent SARS-CoV-2 infection, both the European Society for Medical Oncology (ESMO) and the National Comprehensive Cancer Network (NCCN) recommend an individualized approach to chemotherapy [22,23]. In patients with hematological malignancies, the National Comprehensive Cancer Network (NCCN) recommends delaying therapy until all symptoms have resolved and at least 20 days have passed since symptom onset. In asymptomatic individuals, treatment should be deferred for a minimum of 10 days following the first positive RT-qPCR test for SARS-CoV-2 RNA, provided they remain symptom-free. However, if urgent cancer treatment is required due to uncontrolled disease progression, chemotherapy or other oncologic interventions may be initiated at the discretion of the attending physician without delay [22]. The latest ECIL-10 recommendations advocate for early antiviral treatment with nirmatrelvir/ritonavir or remdesivir in symptomatic hematooncological patients. In cases of severe or critical COVID-19, the use of dexamethasone, interleukin-2 or interleukin-6 inhibitors, or Janus kinase inhibitors is recommended, alongside optimal supportive and intensive care. In asymptomatic patients, the deferral of intensive chemotherapy, conditioning regimens, or cell therapies should be considered on a case-by-case basis [24]. However, the above-mentioned recommendations do not provide guidance on the management of hematological patients with concurrent SARS-CoV-2 and HIV infections. Individualizing therapy in this group is particularly challenging due to the lack of supporting data.

In the case of the patient treated at our facility, persistently positive SARS-CoV-2 test results led to the postponement of anti-lymphoma therapy. Nevertheless, treatment was ultimately initiated despite ongoing SARS-CoV-2 positivity, given that untreated aggressive lymphomas, such as HIV-associated DLBCL, are invariably fatal. This approach aligns with the most recent EHA-ESMO Guidelines for HIV-associated lymphomas published in 2024, which were not yet available at the time of the patient’s management. These guidelines recommend treatment regimens analogous to those used in HIV-negative individuals [25]. In our patient, however, the intensity of chemotherapy was reduced by omitting rituximab, due to its well-documented depletion of normal CD20-expressing B cells for several months [26]. Rituximab and other anti-CD20 monoclonal antibodies were initially associated with increased mortality in patients with COVID-19 [27,28]; however, more recent studies have failed to confirm this association [6,29].

As a result of multiple infections, including COVID-19, the relative dose intensity (RDI) of chemotherapy was suboptimal, leading to progression of the underlying disease. Given the inability to maintain an adequate RDI, a decision was made to discontinue further chemotherapy.

The patient was treated with molnupiravir for COVID-19. Molnupiravir has been shown to be effective in reducing the risk of hospitalization or death in at-risk, unvaccinated adults with COVID-19 [30]. In a multicenter analysis by Bolkun et al., none of the patients treated with molnupiravir died because of hematologic malignancy progression due to the discontinuation of therapy during severe COVID-19 [31]. This stands in contrast to the present case, in which molnupiravir was insufficient to prevent a poor lymphoma-related outcome. This is likely attributable to profoundly impaired immunocompetence and the presence of multiple severe concomitant infections.

In the present case, the patient remained SARS-CoV-2 positive for at least 113 days. He tested positive both on antigen assays, with a specificity estimated at approximately 99.5% [32] and on RT-qPCR tests with low cycle threshold (Ct) values, indicating a high viral RNA load in the upper respiratory tract. Although cell culture—considered the gold standard for detecting viable, replication-competent virus [33]—was not performed, the clinical course of COVID-19 combined with laboratory findings strongly suggests that the patient remained highly contagious throughout hospitalization.

In summary, our case highlights how prolonged SARS-CoV-2 infection may critically impair the ability to deliver optimal chemotherapy, ultimately worsening lymphoma outcomes. However, the presented case should be interpreted as an individual example illustrating the complexity of treating lymphoma in the setting of HIV infection and COVID-19, rather than as a basis for broad conclusions. Moreover, patient’s poor outcome was likely the result of multiple factors, including HIV-associated immunosuppression and opportunistic infections, in addition to prolonged SARS-CoV-2 positivity. The few previously published reports suggest that anti-lymphoma treatment can sometimes be safely administered during ongoing SARS-CoV-2 infection, but data remain very scarce. This underscores the urgent need for further clinical evidence and tailored recommendations to guide treatment decisions in this highly vulnerable group of patients.

## 4. Conclusions

In conclusion, simultaneous occurrence of HIV, lymphoma requiring therapy, and COVID-19 is a therapeutic challenge raising clinical dilemmas regarding the appropriate intensity of anti-lymphoma therapy, as well as the complexities of managing SARS-CoV-2 infection in this population. Although most reported cases with such a co-occurrence were mild, we report a case with fatal outcome due to unavailability to keep relative dose intensity and concomitantly treat superimposable opportunistic and non-opportunistic infections. This case confirms prolonged SARS-CoV-2 shedding and persistently high viral loads in this patients’ population. Without doubt, further data are urgently needed to inform evidence-based treatment strategies for this highly vulnerable group.

## Figures and Tables

**Table 1:** People living with HIV (PLWH) diagnosed with lymphoma and concomitant SARS-CoV-2 infection reported in the literature (ART—antiretroviral therapy; auto-HSCT—autologous hematopoietic cell transplantation; CNS—central nervous system; CRT—chemoradiotherapy; CTx—chemotherapy; HL—Hodgkin lymphoma; CIT—chemoimmunotherapy; IT MTX—intrathecal methotrexate; NA—not available; VGPR—very good partial response; XRT—radiotherapy).

Reference	Age (at Diagnosis of SARS- CoV-2 Infection)	Type of Lymphoma	Anti-Lymphoma Regimen	Last Administration of Anti-Lymphoma Treatment	HIV Infection Management	Concomitant Diseases, Risk Factors of Severe COVID-19	COVID-19 Vaccination	COVID-19 Severity	SARS-CoV-2 Shedding (Days)	COVID-19 Treatment	Hematologic Response at Last Follow-Up
Maan I, et al., 2022 [11]	28	DLBCL	R-CHOP (5 cycles), IT MTX (1 cycle)	Ongoing during SARS-CoV-2 infection	ART temporary interrupted during 5th cycle of CIT	None	NA	Asymptomatic	164	Not required	Remission
Szwebel TA et al., 2020 [14]	NA	B-cell lymphoma	CTx + auto-HSCT, 2 lines CIT + rituximab, 1 line CIT with obinutuzumab + ibrutinib	2 months prior SARS-CoV-2 infection (obinutuzumab and ibrutinib)	ART, undetectable viral load	Lymphopenia, hypogammaglobulinemia	NA	Severe	66	Corticoids, lopinavir/ritonavir, tocilizumab, convalescent plasma	VGPR
Kuczborska K, et al., 2022 [15]	17	Primary CNS B-cell lymphoma	CTx + XRT	Ongoing XRT	No ART before COVID-19	Cachexia, drug-induced nephropathy	No	Mild	NA	Remdesivir	NA
Mardani M, et al., 2021 [16]	61	HL	CTx + CRT + auto-HSCT	Auto-HSCT 2 months prior SARS-CoV-2 infection	ART, undetectable viral load	Underweight	NA	Mild	NA	Atazanavir	NA
Arbune M, et al., 2025 [17]	57	HL	NA	NA	darunavir/cobicistat/emtricitabine/tenofovir alafenamide	Hypoalbuminemia	No	Severe	NA	Remdesivir	NA

**Table 2:** The results of SARS-CoV-2 tests obtained from the described patient (Ct—cycle threshold; RAT—rapid antigen test; RT-qPCR—reverse-transcription real-time polymerase chain reaction); “+”—positive.

Day	Test	Result
1	RT-qPCR	+, Ct: 27 (gene N), 20 (gene RdRP)
9	RAT	+
14	RAT	+
18	RAT	+
21	RAT	+
23	RAT	+
25	RAT	+
29	RAT	+
36	RT-qPCR, RAT	+, Ct: 21 (gene N1), 21 (gene N2)
43	RAT	+
50	RT-qPCR, RAT	+, Ct: 17 (gene N1), 19 (gene N2)
57	RAT	+
65	RT-qPCR, RAT	+, Ct: 11 (gene N1), 11 (gene N2)
71	RAT	+
77	RAT	+
78	RT-qPCR	+, Ct 11 (gene ORF1), 10 (gene ORF8), 10 (gene N)
85	RAT	+
92	RT-qPCR, RAT	+, Ct 13 (gene ORF1), 14 (gene ORF8), 16 (gene N)
99	RAT	+
106	RAT	+
113	RAT	+

## Data Availability

No new data were created or analyzed in this study. Data sharing is not applicable.
